# Sleep Quality and Cognitive Decline Across the Adult Age Range: Findings from the Maastricht Aging Study (MAAS)

**DOI:** 10.3233/JAD-230213

**Published:** 2023-11-21

**Authors:** Sebastian Köhler, Lion M. Soons, Huibert Tange, Kay Deckers, Martin P.J. van Boxtel

**Affiliations:** a Department of Psychiatry and Neuropsychology, School for Mental Health and Neuroscience (MHeNs), Maastricht University, Maastricht, The Netherlands; b Alzheimer Center Limburg, Maastricht University Medical Center+, Maastricht, The Netherlands; c Department of Family Medicine, Care and Public Health Research Institute (CAPHRI), Maastricht University, Maastricht, The Netherlands

**Keywords:** Adult, aging, Alzheimer’s disease, cognitive function, lifestyle, risk factors, sleep quality

## Abstract

**Background:**

Sleep disturbances have been linked with cognitive decline and a higher risk of dementia. However, there is a lack of studies with sufficient follow-up duration, a detailed neuropsychological assessment and adequate control of main confounders.

**Objective:**

To investigate the relation between self-reported sleep quality and cognitive decline over 12 years in cognitively healthy individuals from the general population.

**Methods:**

We used data from the Maastricht Aging Study (MAAS), a Dutch population-based prospective cohort study of 1,823 community-dwelling adults aged 24 to 82 years at baseline. Cognitive performance was measured at baseline, 6 and 12 years on verbal memory, executive functions, and information processing speed. Sleep quality was assessed at baseline using the sleep subscale score of the 90-item Symptom Checklist (SCL-90). Additional modifiable dementia risk factors were summarized in the LIfestyle for BRAin health (LIBRA) risk score. Weighted linear mixed models tested the association between continuous scores and tertiles of subjective sleep quality and change in cognitive performances over time. Models were adjusted for age, gender, educational level, LIBRA, and use of hypnotic (sleep) medication.

**Results:**

Worse sleep quality was associated with faster decline in processing speed. At older age (≥65 years), it was also associated with faster decline in verbal memory. Association were independent of other modifiable dementia risk factors and use of hypnotic medication. Directionally similar but non-significant associations were found between worse sleep quality and executive functions.

**Conclusions:**

In this population-based study across the adult age range, poor self-reported sleep was associated with accelerated cognitive decline.

## INTRODUCTION

Dementia, characterized by progressive cognitive and functional decline, is a global health problem affecting about 57 million people worldwide [1]. With no cure so far, targeting modifiable risk and protective factors may lead to effective risk reduction strategies [2], with proven modest effects on cognitive performance over time [3]. Several modifiable risk factors for dementia have been identified including diet, physical and cognitive activity, and vascular conditions [4], accounting for approximately 40% of the total risk [5]. The remaining 60% might be explained by non-modifiable risk factors (e.g., age, gender, genetics), gene-environment interactions, as well as additional, yet under-investigated modifiable risk factors.

In a rapidly evolving modern society, an increasing number of people suffer from sleep problems that have in turn been associated with a range of health problems, including circadian rhythm disruption, depression, anxiety, increased use of alcohol and drugs and medical conditions [2, 6–9]. Previous studies that examined the association between subjective sleep problems and cognition have shown mixed results. Such studies often focused on specific populations, such as people with dementia or mild cognitive impairment, or community dwelling old to very old individuals [10–12], had a short follow-up duration [13] or had limited cognitive outcome assessment [12, 14, 15]. In a cross-sectional study of older adults (aged 50 years and over), worse self-reported sleep quality has been associated with lower levels of cognitive functioning [16]. In prospective studies, several sleep characteristics have been associated with worse cognitive functioning over time. For example, in a longitudinal study, frequent snoring, higher daytime sleepiness and longer sleep duration at older age (baseline age 63±8 years) was related to faster decline in executive function but not in memory or processing speed during an average follow-up of six years [17]. Another study showed that self-reported fair and poor sleep quality and short and long durations of sleep were associated with lower cognitive scores over a four-year follow-up period [15].

In the Rotterdam Study, no association between self-reported sleep quality and dementia risk was observed [18]. In contrast, a meta-analysis of eighteen longitudinal studies with 246,786 participants in total found that people with diverse self-reported sleep disturbances, including insomnia, sleep disordered breathing, and other sleep problems, had a higher risk of incident all-cause dementia, Alzheimer’s disease (AD), and vascular dementia after an average follow-up of 9.5 years [19]. Self-reported sleep disturbances were associated with a marginally increased risk for all-cause dementia (relative risk (RR) = 1.08, 95% CI: 1.00–1.16), while self-reported insomnia itself was not (RR = 1.17, 95% CI: 0.95–1.43). Similarly, another meta-analysis of 51 longitudinal studies found evidence supporting ten types of self-reported sleep conditions including insomnia, sleep fragmentation, daytime dysfunction, prolonged latency, rapid eye movement sleep disorder, excessive time in bed, apnea, no habitual napping, sleep inefficiency, and increased sleep duration as predictors of higher risk of all-cause cognitive disorders [20]. In both meta-analyses, heterogeneity among studies was high, and the need to further understand heterogeneity in findings and possible meditators and confounders of the observed association, such as the role of depression, was highlighted [19]. In addition, the majority of studies generally focused on older adults (≥65 years), and heterogeneity by age and gender has not been well elucidated.

Taken together, there is a lack of studies with sufficient follow-up duration, a detailed neuropsychological assessment and adequate control of main confounders to understand the association between sleep quality and cognitive decline in cognitively healthy individuals. We therefore aim to investigate whether self-reported sleep quality is associated with cognitive decline over a 12-year follow-up period in a general population sample across the full adult age range. Furthermore, we aim to explore differences by age (younger (< 65 years) and older adults (≥65 years)) and by gender. We hypothesize that participants with lower self-reported sleep quality show more cognitive decline after 12 years compared to participants with higher self-reported sleep quality.

## METHODS

### Study population

The Maastricht Aging Study (MAAS) started in 1993 as a prospective cohort study into normal and pathological cognitive aging. A random selection of 10,396 people were sampled from the Registration Network Family Medicine (RNFM) of collaborating general practitioners in the south of the Netherlands [21]. As each person in the Netherlands is registered with a general practitioner, the RNFM is considered representative for the general population. Exclusion criteria were medical conditions known to interfere with normal cognitive function: cerebrovascular pathology, nervous system tumor or congenital malformation, multiple sclerosis, Parkinsonism, epilepsy, dementia, organic psychosis, schizophrenia, affective psychosis, and intellectual disability. Next, a stratified random sample based on age, gender, and occupational level (low/high) took part in a longitudinal phase of the study (*n* = 1,823, age range 24 to 82 years). The participants underwent a comprehensive assessment of medical status, lifestyle, anthropomorphic and neuropsychological measures at baseline, 6 and 12 years of follow-up. The study was approved by the local medical ethics committee of Maastricht University Medical Centre (MEC05-107). All participants gave informed consent.

### Subjective sleep quality

Sleep quality at baseline was assessed with the sleep subscale of the Dutch version of the Symptoms Check List-90 (SCL-90) [22]. This subscale includes three items, related to ‘prolonged sleep latency/problems falling asleep’, ‘early awakening’, and ‘disturbed or restless sleep’ and has been shown to be an acceptable screening tool for sleep problems when compared to other commonly used tools [23]. Items were scored on a 5-point Likert scale, ranging from ‘1 = not at all’ to ‘5 = very much’, which were summed up in a total score ranging from 3 (no problems) to 15 (many problems). SCL-90 sleep subscale scores were binned into tertiles to dummy code for three levels labelled ‘good’ (tertile 1), ‘average’ (tertile 2), and ‘poor’ (tertile 3) sleep quality.

### Assessment of cognitive functions

Trained psychological assistants administered a set of neuropsychological tests to assess cognitive function at baseline and at 6- and 12-year follow-up in the domains of verbal memory, executive function, and information processing speed. For the former, the visual Verbal Learning Test (VLT) was used [24]. The test consists of 15 non-related words (monosyllabic), which are shown on a computer screen in 5 subsequent trials. After each trial, a recall phase follows (immediate recall). After 20 min, participants are asked to spontaneously recall the words (delayed recall), followed by a recognition phase of old and new words (delayed recognition). In the present study, the delayed recall score was used as domain outcome. Executive functions were measured with The Concept Shifting Test (CST) [25]. This is a timed task that asks participants to cross out ascending digits (part A) and letters (part B). In the final trial, participants have to alternate between digits and letters as fast as possible (part C). The outcome in the present study was the shifting score on this task (measuring divided attention and mental flexibility), which is the differences between time needed for completing part C minus time for (part A + part B) /2 (i.e., the average). This score was the domain outcome in our study. To measure information processing speed, the Letter Digit Substitution Test (LDST) was used. Participants have to match digits and letters according to a key on a response sheet as quickly as possible within 90 s [26]. The number of correct responses was the domain outcome.

### Potential confounders and modifiable risk factors

Age (in years), gender (male/female), education level (low, middle, high), and marital status (single, married or living with partner, divorced, widowed) were included as demographic covariates. Use of hypnotic (sleep) medication was assessed at baseline by self-report. Dementia status was ascertained after 12 to 15 years, based on information from MAAS combined with the continuous morbidity registration in the RNFM. Additional health and lifestyle-related risk factors for dementia were combined in the LIfestyle for BRAin health (LIBRA) index. LIBRA summarized 12 modifiable or manageable dementia risk factors in a weighted sum score, with weights based on a factor’s relative risk in meta-analyses [4]. It has been used in different prospective cohorts and multifactorial trails for prediction of cognitive decline and dementia [27–34]. Questionnaires, anthropomorphic measures, and clinical information were used to assess presence of 11 risk and protective factors. Self-reported physical inactivity was based on hours up and about (lowest tertile was considered at risk) or exercising < 150 min/week (risk weight = +1.1). Current smoking (risk weight = +1.5) and alcohol intake≤7 consumptions/week (protective weight = –1.0) were self-reported. Presence of obesity was based on a body mass index (BMI) ≥30 kg/m^2^ or, if missing, a waist circumference of > 88 cm and > 102 cm for women and men, respectively (risk score = +1.6). Presence of depression was assessed with the Symptom Check List (SCL-90) subscale (highest quartile received a risk score = +2.1). Hypertension was assessed by an average office systolic blood pressure ≥140 mmHg, a diastolic blood pressure ≥90 during 5 serial assessments, use of antihypertensives or, if all missing, self-report (risk weight = +1.6). Presence of type-2 diabetes (risk weight = +1.3), hypercholesterolemia (risk weight = +1.4), heart disease (risk weight = +1.0), or chronic kidney disease other than kidney stones (risk weight = +1.1) was based on self-reported medical diagnosis or use of medication. Cognitive activity (protective weight = –3.2) was based on whether people fell in the highest tertile of hours/week spent on reading books, magazines, newspapers, or playing mind games (chess, checkers, puzzles), divided into tertiles (low/medium/high). There was no data in MAAS on adherence to the Mediterranean diet (protective weight = –1.7). Final LIBRA scores at baseline ranged from –4.2 to +9.2, with higher scores indicating higher dementia risk.

### Statistical analyses

To investigate baseline differences between sleep quality tertiles (poor, average, good sleep quality), we used ANOVA’s and χ^2^-tests. The VLT delayed recall score was square root transformed to correct for a non-normality. To test the crude and adjusted associations between baseline sleep quality scores and cognitive decline from baseline (F0) to 6-year (F1) and 12-year follow-up (F2), linear-mixed model (LMM) analysis was used. Advantages of LMM over ANOVA-based repeated measures include that it does not use listwise deletion, as it handles missing observations by using maximum likelihood to estimate missing values conditional on covariates [35]. To model the associations between sleep quality and changes in cognition, the interaction of sleep score-by-time was included, and the overall interaction was tested using a Wald *χ*^2^-test (2 degrees of freedom). Likelihood ratio tests suggested to include random intercepts and random slopes with an unstructured covariance matrix. Terms for the fixed effects in Model 1 included the intercept, sleep quality score, time (2 dummies), age, gender, education (low, middle, high), and the interaction term between time and sleep. In Model 2, the LIBRA score and its interaction with time was added to Model 1 to test whether sleep explains additional variance in the cognition outcomes over and above other modifiable dementia risk and protective factors. In the final Model 3, use of hypnotic (sleep) medication was added to Model 2. Also, we used inverse probability weights to reduce selection bias: 1) a sampling weight to generalize estimates to the RNFM source population from which the participants were drawn, and 2) an attrition weight for an individual’s propensity of being lost to follow-up [36]. To test differences by age groups (<65 years, *n* = 1,333; ≥65 years, *n* = 490) and gender, the respective three-way interactions with continuous sleep scores and time were included, followed by stratified analysis if interactions were statistically significant. To test for non-linear associations, the models with continuous sleep scores were compared to the models with sleep tertiles by likelihood ratio testing. To reduce the possibility for reverse causation, in which sleep problems are early signs of prodromal dementia, we ran additional analyses excluding participants who developed dementia during the follow up period. *P*-values < 0.05 were considered statistically significant in two-sided tests. All analyses were performed using Stata 17 (StataCorp, TX)

## RESULTS

### Baseline differences

From the 1,823 participants, 49 had missing data on subjective sleep quality at baseline 2 had > 10 LIBRA factors, and 5 had missing data on marital status. They were excluded from analyses. Of the remaining 1,767 participants, 543 (30.7%) individuals were classified with having poor sleep quality at baseline. Baseline sample characteristics, stratified by tertiles of sleep quality status, are shown in Table 1. On average, they were older, lower educated, more often female, less often married, and had higher (i.e., unhealthier) LIBRA scores compared to individuals with good sleep quality.

**Table 1:** Baseline characteristics of the study sample and differences by subjective sleep quality tertiles

	Total study population	Subjective sleep quality		
Characteristic	*n* = 1,767	Good (*n* = 726)	Average (*n* = 498)	Poor (*n* = 543)	*p*
Age, mean±SD	51.0±16.3	48.2±16.2	51.4±16.6	54.5±15.4	<0.001
<65 y	1,318 (74.6)	572 (78.8)	366 (73.5)	380 (70.0)	0.001
≥65 y	449 (25.4)	154 (21.2)	132 (26.5)	163 (30.0)
Female, *n* (%)	877 (49.6)	302 (41.6)	263 (52.8)	312 (57.5)	<0.001
Education, *n* (%)					<0.001
Low	623 (35.3)	209 (28.8)	182 (36.6)	232 (42.7)
Middle	736 (41.6)	336 (46.3)	190 (38.1)	210 (38.7)
High	408 (23.1)	181 (24.9)	126 (25.3)	101 (18.6)
Marital status, *n* (%)					<0.001
Single	186 (10.5)	88 (12.1)	55 (11.1)	43 (7.9)
Married or living with partner	1,395 (79.0)	596 (82.1)	386 (77.5)	413 (76.1)
Divorced	52 (2.9)	15 (2.1)	15 (3.0)	22 (4.0)
Widowed	134 (7.6)	27 (3.7)	42 (8.4)	65 (12.0)
LIBRA score^a^, mean±SD	0.8±2.3	0.5±2.1	0.6±2.4	1.4±2.4	<0.001
Verbal memory^a^, mean±SD	9.7±3.0	9.9±3.0	9.8±3.1	9.5±2.9	0.073
Executive functions^b^, mean±SD	11.9±10.8	10.9±8.9	12.2±11.5	12.9±12.5	0.003
Information processing speed^c^, mean±SD	48.7±11.6	50.1±11.4	48.6 ±11.8	46.9±11.4	<0.001

^a^Delayed recall of the verbal learning test (raw); higher scores indicate better performance. ^b^Shifting score of the CST (raw); lower scores = better cognition. ^c^LDST (raw); higher scores = better cognition. LIBRA, LIfestyle for BRAin health score; SD, standard deviation.

### Baseline sleep quality and cognitive decline

Results for sleep scores at baseline and change in cognition during the 12-year follow-up are presented in Table 2. At baseline, no associations were found between subjective sleep quality and cognitive performance. In the full-adjusted Model 3, significant sleep scores-by-time interactions showed lower subjective sleep quality was associated with faster decline in verbal memory (*χ*^2^ = 8.69, df = 2, *p* = 0.013). While participants without sleep problems (score = 0) showed a significant increase in verbal memory from baseline to 6 years (B = 26.50, 95% CI = 20.06;32.94, *p* < 0.001) and baseline to 12 years (B = 22.26, 95% CI = 14.09;30.43, *p* < 0.001), this was significantly attenuated by –1.70 and –2.08 points, respectively, with every additional score on the sleep problem scale (Table 2).

**Table 2:** Associations between continuous sleep quality scores with baseline cognition and change in cognition

	Baseline difference per		Additional change from		Additional change from	
	unit increase in sleep		baseline to 6-years per unit		baseline to 12-years per unit	
	quality scores		increase in sleep quality scores		increase in sleep quality scores	
Test Battery	Estimate^a^	95% CI	Estimate^b^	95% CI	Estimate	95% CI
*Verbal memory*
Model 1	0.24	–0.72 to 1.21	–1.88^*^^*^	–3.15 to –0.61	–2.13^*^	–3.80 to –0.46
Model 2	0.36	–0.62 to 1.35	–1.71^*^^*^	–2.96 to –0.45	–2.09^*^	–3.76 to –0.42
Model 3	0.34	–0.64 to 1.32	–1.70^*^^*^	–2.96 to –0.45	–2.08^*^	–3.75 to –0.42
Age < 65 y	–0.32	–1.50 to 0.86	–0.46	–1.61 to 0.69	–0.72	–2.21 to 0.77
Age≥65 y	1.02	–0.69 to 2.73	–3.64^*^^*^	–5.98 to –1.29	–3.48	–7.92 to 0.96
*Executive function*
Model 1	–0.09	–0.33 to 0.16	0.13	–0.32 to 0.57	0.30	–0.33 to 0.93
Model 2	–0.14	–0.39 to 0.11	0.14	–0.28 to 0.55	0.34	–0.24 to 0.93
Model 3	–0.14	–0.39 to 0.11	0.14	–0.28 to 0.55	0.34	–0.24 to 0.93
*Information processing speed*
Model 1	0.08	–0.10 to 0.27	–0.18	–0.37 to.002	–0.40^*^^*^	–0.66 to –0.14
Model 2	0.15	–0.04 to 0.33	–0.16	–0.35 to 0.03	–0.35^*^^*^	–0.61 to –0.09
Model 3	0.15	–0.04 to 0.33	–0.16	–0.35 to 0.03	–0.35^*^^*^	–0.62 to –0.09

^a^Estimates are unstandardized regression coefficients showing difference in baseline cognition per unit decrease in Symptom Checklist-90 sleep quality score. ^b^Estimates are unstandardized regression coefficients showing additional change in cognition from baseline to year 6 or year 12 per unit decrease in Symptom Checklist-90 sleep quality score relative to those without sleep problems. Model 1: Age, gender, education, marital status; Model 2: Model 1 + LIfestyle for BRAin Health score (LIBRA); Model 3: Model 2 + use of hypnotic (sleep) medication. CI, confidence interval; ^*^p < 0.05, ^*^^*^p < 0.01, ^*^^*^^*^p < 0.001. For verbal memory and information processing speed, higher scores indicate better performance. For executive function, lower scores indicate better performance. Scores for verbal memory are square-transformed for approximating a normal distribution.

For information processing speed, a faster decline with increasing sleep problems was observed (*χ*^2^ = 7.21, df = 2, *p* = 0.027). Participants with excellent sleep showed an increase in scores from baseline to 6 years (*B* = 2.61, 95% CI = 1.71;3.52, *p* < 0.001) and a non-significant decline from baseline to 12 years (*B* = –0.90, 95% CI = –2.20;0.40, *p* = 0.174), these change scores declined by –0.16 and –0.35 points with very additional score on the sleep problem scale.

Directionally similar but statistically non-significant associations were observed for executive functions. Comparing Model 3 with Model 1, estimates of decline were similar, suggesting that associations do not change after correcting for LIBRA and hypnotic (sleep) medication. Also, additional adjustment for marital status did not change associations.

### Effect modification by age and gender

Next, we tested interactions by age-groups and gender (Model 3). Age modified the association between subjective sleep quality and cognitive decline in verbal memory (*χ*^2^ = 10.88, df = 2, *p* = 0.004). Age-stratified analyses clarified this by showing that in adults≥65 years at baseline, but not younger ones, lower sleep quality was associated with faster decline in verbal memory (*χ*^2^ = 10.17, df = 2, *p* = 0.006). Testing of three-way interactions showed no effect modification by gender for the association between subjective sleep quality and cognitive decline.

### Additional analyses

Comparing the models with continuous sleep scores to the models with sleep tertiles suggested no deviation from the linearity assumption (results not shown). To explore whether the effect of sleep problems might be a prodromal symptoms of dementia progression, we excluded cases with incident dementia (*n* = 62) during the 12-year follow-up. The associations remained significant, suggesting that prodromal dementia cases were unlikely to drive the association between sleep quality and cognition. Finally, we tested whether we should adjust for age-dependent changes in the modifiable risk factors summarized by LIBRA. For this, we included the 3-way-interactions of LIBRA x Time x Age in Model 3. These 3-way interactions were not statistically significant.

## DISCUSSION

The present study investigated the association between sleep quality and decline in cognition over a period of 12 years in adult individuals aged 24 to 82 years. In the total sample, those who reported worse sleep quality showed faster decline in information processing speed. In addition, people aged≥65 years with worse sleep quality had a faster decline in verbal memory. Associations were robust after adjusting for a range of covariates, including hypnotic (sleep) medication use and a modifiable dementia risk score. No modifying effect of gender was observed.

Our results are supported by previous meta-analyses, showing that self-reported sleep problems are associated with a higher risk of dementia [19, 20]. In the Northern Manhattan Study, snoring, daytime sleepiness, and sleep duration were associated with faster decline in executive function after adjusting for demographic characteristics and a vascular risk score in 687 adults (mean age 63 years), but not with change in episodic memory, language, or processing speed [17]. No association between subjective sleep quality and dementia risk was found in the Rotterdam Study [18]. Notably, this cohort was much older (mean age 72 years) than participants in MAAS (mean age at baseline 52 years), which suggests that the effects of sleep quality on the brain pathology and functional outcomes are age dependent.

The broad age range in MAAS allowed us to explore such heterogeneity by age. Interactions analyses showed that older adults (≥65 years), relative to younger adults (<65 years), also experienced faster decline in verbal memory, which might indicate a segregation at the pathological level. In a cohort enriched for parental history of late-onset AD, poor sleep quality in 101 adults (mean age 63 years) was associated with lower cerebrospinal fluid (CSF) Aβ_42_/Aβ_40_ and higher t-tau/Aβ_42_, p-tau/Aβ_42_, MCP-1/Aβ_42_, and YKL-40/Aβ_42_ indicating higher AD pathology [37]. However, reverse causality, in which underlying brain pathology caused sleep disturbance and memory impairment, cannot be ruled out, despite the 12-year longitudinal design. Presence of white matter hyperintensities in the brain affects sleep quality and contributes to sleep difficulties, at least in patients with heart failure [38]. Moreover, age-related decline in sleep duration can be explained by atrophy of cerebral grey matter and incipient AD [39].

Previous studies that examined different sleep architectures with objective measurements including polysomnography [40], actigraphy [41], blood pressure [42], or diffusion tensor imaging and magnetic resonance imaging scans [43] also showed an association between sleep architecture and cognitive function or dementia risk. Taken together, studies thus suggest that both objective and subjective evidence of sleep dysfunction impact on brain health. The latter is more easily assessed in clinical routine and might thus be more easily implemented in dementia risk reduction measurements.

Our study has several strengths and limitations. We included a relatively large sample across a wide age range allowing for age-dependent analyses. The observational period was sufficient for studying cognitive decline, with 6- and 12-year cognitive re-assessments, and use of a comprehensive neuropsychological test battery covering three major cognitive domains. Although additional cognitive tests were administered, the reported cognitive variables have been shown to be sensitive to age-related cognitive change and associated with other risk factors like hypertension [36], diabetes [44], and cardiovascular disease [45]. Consistent use allows for comparability of findings across reports, while using a restricted set of (sensitive) outcomes reduces the number of statistical tests and, thus, the chance of type 1 error. Finally, the statistical methods fully exploited the longitudinal design of MAAS while controlling for several relevant covariates. However, there are also limitations. Selection bias might have occurred by losing participants who were older, frailer, and with more medical conditions, which may have resulted in an underestimation of the association between sleep quality and cognitive decline. Sleep quality was assessed by self-report, while objective measures such as polysomnography or actigraphy could have provided additional, more objective information about sleep.

In conclusion, this study indicates that adults with worse subjective sleep quality showed accelerated cognitive decline. Findings point towards an opportunity for prevention of sleep-related cognitive abnormalities though raising awareness on the importance of good sleep quality for healthy brain aging during the adult lifespan.

## Data Availability

The data supporting the findings of this study are available on request from the corresponding author. The data are not publicly available due to privacy or ethical restrictions.
